# Production of recombinant intact and N-terminal truncated lipoxygenase isozyme III expressed in *Saccharomyces cerevisiae* and its influence on glutenin polypeptides

**DOI:** 10.1016/j.fochms.2024.100195

**Published:** 2024-01-30

**Authors:** Shunsuke Takahashi, Gao Yue, Reina Miyagi, Shiiba Kiwamu

**Affiliations:** Division of Life Science and Engineering, School of Science and Engineering, Tokyo Denki University, Ishizaka, Hatoyama-cho, Hiki-gun, Saitama 350-0394, Japan

**Keywords:** Lipoxygenase, Glutenin, Wheat flour, Dough

## Abstract

This study investigated the effects of wheat lipoxygenase isozyme III (LOX III) and its truncated form, Mini-LOX III, on flour dough properties using yeast-expressed recombinant enzymes and hypothesized their potential to enhance cereal-based food quality. These enzymes actively catalyze linoleic acid, which is crucial for dough formation. The addition of recombinant LOX III and Mini-LOX III to wheat flour significantly changed glutenin protein composition. An increase in the amount of soluble glutenin and a shift in polypeptide distribution were observed, marked by a decrease in the high-molecular-weight regions and an increase in the low-molecular-weight regions. This result reflects the role of enzymes in altering the hydrophobicity of glutenin surfaces, thereby affecting the protein solubility and dough properties. Thus, recombinant LOX III and Mini-LOX III offer new avenues for enhancing the texture and quality of cereal-based foods, providing valuable insights into the role of wheat LOX in flour processing and its potential industrial applications.

## Highlights

• Production of yeast-expressed wheat lipoxygenase isozyme III (LOX III) and Nterminal truncated LOX III isozyme (Mini-LOX III) was established.
• Wheat recombinant LOX III and Mini-LOX III extracted from yeast acted as linoleic acid substrates.
• The polypeptide composition of soluble glutenin was changed by adding wheat recombinant LOX III or Mini-LOX III.

## 1 Introduction

Lipoxygenase (LOX, EC 1.13.11.12) catalyzes the oxidation of polyunsaturated fatty acids containing *cis*,*cis*-1,4-pentadiene. However, in studies of rheological effects on flour dough, soybean LOX has been shown to mediate the oxidation of sulfhydryl groups in dough protein, resulting in structural changes in the proteins (Daniels et al., 1970, Faubion and Hoseney, 1981, Permyakova and Trufanov, 2011). Additionally, the addition of soybean LOX enhances dough mixing tolerance through interactions with lipids in the dough (Hoseney et al., 1980, Frazier et al., 1977). These findings suggest that LOX significantly affects the quality and texture of cereal foods.

Soybean flour and partially purified soybean LOX have been used as models for understanding biological role of LOX in secondary processing of flour (e.g., bread making). LOX is also present in wheat germ as three isoforms: LOX I, LOX II, and LOX III. In our previous studies, natural LOX isozymes isolated from wheat germ induced a change in glutenin subunit composition by reducing acetic acid-soluble proteins during dough fermentation, resulting in an increase in glutenin proteins in the 70 % ethanol-soluble fraction (Shiiba et al., 1990). These results suggest that wheat LOX isozymes decrease the hydrophobicity of glutenin protein surfaces. In a subsequent study, we evaluated the effects of purified wheat germ LOX I, II, and III on dough production (Shiiba et al., 1991). The dough with wheat LOX had less mixing resistance on the microgram scale than that with soybean LOX. The protein SH/SS ratio in dough with wheat LOX isozymes was approximately 20 % lower than that without wheat LOX. Additionally, focusing on the differences in wheat LOX isozymes, flour with LOX III had a stronger effect on the dough’s bread-making quality than dough with either LOX I or LOX II. In particular, in the composition of glutenin subunits in the flour containing the wheat LOX III, we observed a simultaneous reduction in the content of fraction 1 (which constitutes the aggregating polypeptide of glutenin) and fraction 3 (comprising the low molecular weight subunit of glutenin). This was accompanied by an increase in the quantity of polypeptides in the 70 % ethanol soluble fraction. However, wheat germ contains very little wheat LOX III, which makes it difficult to isolate this enzyme in large quantities. Therefore, it remains unclear how LOX III affects the composition of glutenin subunits.

To solve this problem, we attempted to express recombinant wheat LOX III using a heterologous protein expression system in *Escherichia coli (E. coli)*. Nevertheless, recombinant LOX III, which has a high molecular weight (∼105 kDa), was not expressed in *E. coli*. In contrast, Zhang et al. (2012) successfully expressed recombinant LOX from *the Anabaena* sp. PCC 7120 (ana-rLOX) using a heterologous protein expression system in *Bacillus subtilis* (*B. subtilis*). Dough treated with ana-rLOX improved both whiteness and loaf volume, thereby enhancing the quality of bread and noodle production. Additionally, tryptic digestion of soybean LOX-1 is known to produce a 60-kDa fragment, termed Mini-LOX, which has been shown to enhance both catalytic efficiency and membrane-binding ability compared to the natural LOX-1 enzyme (Maccarrone, et al., 2001).

Here, we established a recombinant LOX III and N-terminal truncated LOX III, which contains only the C-terminal domain (Mini-LOX III) expression system from wheat, using a heterologous protein expression system in the yeast species *Saccharomyces cerevisiae* (*S. cerevisiae*). We evaluated the activities of recombinant LOX III and Mini-LOX III extracted from yeast, using linoleic acid as a substrate. We used the results of this evaluation to assess the effects of recombinant LOX III and Mini-LOX III on the composition of insoluble glutenin polypeptides and to show the polypeptides most susceptible to the oxidative effects of enzymes.

## 2 Materials and methods

### 2.1 Materials and reagents

PrimeSTAR Max DNA Polymerase and Ex-Taq HS DNA polymerase were purchased from Takara (Tokyo, Japan). Primers were ordered from Eurofins Genomics (Tokyo, Japan) and Integrated DNA Technologies (IDT; Coralville, IA, USA). The DNA ligation Kit and JM109 chemically competent cells were purchased from Takara. *S. cerevisiae* BY4741 (BY23849) strain and pGK423 vector (Ishii et al., 2009) were obtained from the National BioResource Project (NBRP)-yeast. All restriction endonucleases were purchased from New England Biolabs (NEB, Ipswich, MA, USA). Growth medium was purchased from Becton Dickinson (BD, Franklin Lakes, NJ, USA). All other reagents were of biotechnology grade and purchased from Bio-Rad (Hercules, CA, USA) and Nacalai Tesque (Kyoto, Japan).

### 2.2 Preparation of plasmids

The constructed plasmids (Table S1) were transformed into JM109 chemically competent *E. coli* (Takara). Transformants were grown on LB agar plates supplemented with the appropriate antibiotics at 37 °C. Positive clones were screened by colony PCR and target plasmids were purified using a QIAprep Miniprep Kit (Qiagen, Tokyo, Japan). The plasmids were then sequenced using the primers listed in Table S2 by GENEWIZ (Azenta Life Sciences, Tokyo, Japan).

### 2.3 Construction of expression vector for wheat LOX III

Codon optimization of the wheat LOX III nucleotide sequence (GenBank HQ913602) for expression in *S. cerevisiae* was assisted by COdon Usage Similarity INdex (COUSIN, https://cousin.ird.fr/index.php) (Bourret et al., 2019), resulting in the coding sequences shown in Table S3. The selected sequences were synthesized by the chemical DNA synthesis company, Twist Bioscience (San Francisco, CA, USA). To construct the pGK423-LOX III vector, synthetic genes were cloned into the pGK423 vector digested with *Sal*I and *Bam*HI. As a LOX III protein expression marker, a synthetic DNA fragment containing a 2A self-cleaving peptide sequence and the fluorescent protein mClover3 gene was cloned via Gibson assembly (Gibson et al., 2009) into the pGK423-LOX III vector via *Eco*RI sites to produce the pGK423-LOX III-2A-FP vector (Fig. S1A). To construct the pGK423-Mini-LOX III vector, the gene truncated at the N-terminus of the full-length LOX III gene (Mini-LOX III) was PCR amplified and cloned into the pGK423 vector digested with *Sal*I and *Bam*HI (Fig. S1B).

### 2.4 Strains and medium

*Saccharomyces cerevisiae* BY4741 (genotype: MATa his3Δ1 leu2Δ0 met15Δ0 ura3Δ0) was used as the host strain. The yeast was transformed using the lithium acetate method (Chen et al., 1992) with pGK423-LOX III-2A-FP and pGK423-Mini-LOX III. Yeast strains were cultivated in synthetic complete dextrose (SCD) medium (6.7 g/L of yeast nitrogen base without amino acids, 2 g/L amino acid dropout mix lacking the appropriate amino acid for selection, and 20 g/L glucose).

### 2.5 Extraction of recombinant LOX III in yeast

Recombinant LOX III expressed in yeast was extracted as follows: 2 mg of yeast was suspended in 5 ml of 50 mM phosphate buffer (pH 7.6) and crushed using a bead crushing device (μT-12; TAITEC, Saitama, Japan). The samples were centrifuged at 20,000xg for 20 min to remove insoluble material, and the supernatant fractions were filtered through 0.22 µm filter membranes. The experimental procedure was also conducted using recombinant Mini-LOX III. To determine the Michaelis constant (Km) value of linoleic acid substrates, the concentration of linoleic acid in this reaction mixture was varied from 10 mM to 70 mM.

### 2.6 Assay of recombinant LOX III and Mini-LOX III activities

Recombinant LOX III activity was determined according to the photometric method (absorbance 234 nm), using linoleic acid as the substrate (Shiiba et al., 1991). Protein determination was carried out according to Lowry’s method, using bovine serum albumin as the standard protein. Under standard assay conditions, one unit of lipoxygenase activity was defined as the quantity of enzyme that generated 1 µmol of conjugated diene per minute. The extinction coefficient for diene was assumed to be 25,000/M/cm per cell (Axelrod et al., 1981). The experimental procedure was also conducted using recombinant Mini-LOX III.

### 2.7 Extraction of glutenin proteins with recombinant LOX

Each 3 ml of water or recombinant LOX III extract (final 0.003 units) was added to 5 g of wheat flour and mixed for 5 min. The dough samples were incubated at 30 °C for 90 min. The prepared dough was homogenized with 50 ml of 100 mM acetate for 10 min and centrifuged at 3500 rpm for 30 min. Ethanol was added to the supernatant solution in 70 % ethanol to precipitate soluble glutenin. The precipitate was dissolved in a solution containing 1 % 2-mercaptoethanol and 0.5 % sodium dodecyl sulfate (SDS). The experimental procedure was also conducted using recombinant Mini-LOX III.

### 2.8 Other methods

Sodium dodecyl sulfate–polyacrylamide gel electrophoresis (SDS-PAGE) was performed on 10 % gels using the standard Laemmli method. Two-dimensional gel electrophoresis (2D-PAGE) was performed as follows: the first dimension (horizontal) was an isoelectric focusing ranging from pH 3 to 11 (left, pH 11; right, pH 3), and the second dimension (vertical) was 12.5 % SDS-PAGE (Maruyama-Funatsuki et al., 2004). Following electrophoresis, the gels were stained using Coomassie Brilliant Blue R-250 (FUJIFUILM Wako Pure Chemical Industries) or an EzStain Silver Kit (Atto, Tokyo, Japan). Protein concentrations were determined using a Bradford assay kit (Bio-Rad, Hercules, CA, USA) with BSA as the standard.

## 3 Results

### 3.1 Characteristics of the wheat LOX III domain

**Fig. 1:**
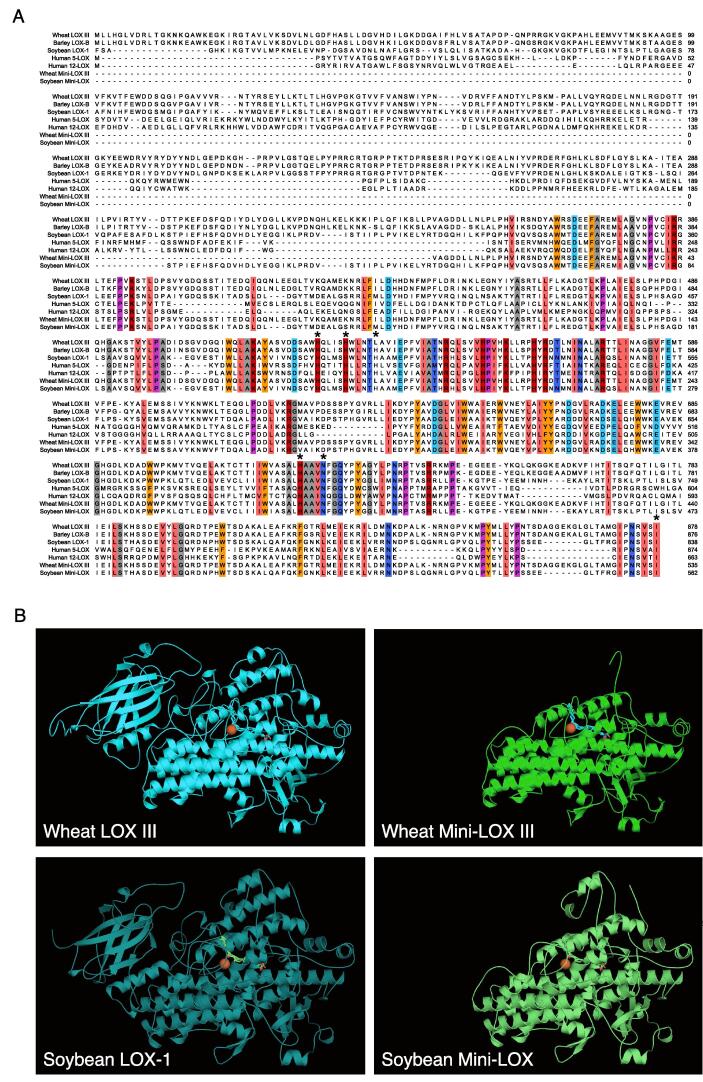
Sequence analysis of lipoxygenase homologs. (A) Multiple sequence alignment was performed using T-Coffee (Notredame et al., 2000) and colored using the Sequence Manipulation Suite (Stothard, 2000) (gray: G, A, V, L, I; scarlet: F, Y, W; orange: M; green: S, T; red: K, R, H; light blue: D, E; blue: N, Q; purple: P) to highlight identical or similar residues according to their biochemical properties for all sequences at a given position. Five amino acid iron ligands are marked with asterisks (Zhang et al. 2007). (B) Three-dimensional structures of wheat and soybean. Wheat LOX III (AlphaFold2 structure prediction (Jumper et al., 2021, Varadi et al., 2022): F1DTB8), wheat Mini-LOX III, soybean LOX-1 (PDB: 1YGE), and soybean Mini-LOX. (For interpretation of the references to colour in this figure legend, the reader is referred to the web version of this article.)

Wheat LOX III is 878 amino acids in length, corresponding to a molecular weight of 95 kDa. The size and structure of wheat LOX III were similar to those of soybean LOX and other cereal LOX enzymes, and their amino acid sequences shared more than 50 % homologous sequence identity (Fig. 1A and B). Soybean LOX-1 is cleaved by trypsin between lysine 277 and serine 278 amino acid residues, separating the N- and C-terminal domains. This C-terminal domain, produced by trypsin digestion of Soybean LOX-1, is known as Mini-LOX. N-terminal amino acid analysis of Mini-LOX has shown that the N-terminal amino acid sequence is STPIEFHSFQ (Maccarrone et al., 2001). Using this sequence, we truncated the N-terminal domain of wheat LOX III and generated the C-terminal domain of wheat LOX III (Mini-LOX III). Mini-LOX III is 536 amino acids long, corresponding to a molecular weight of approximately 60.8 kDa. The size and structure of Mini-LOX III were similar to those of soybean Mini-LOX (Fig. 1A and B) and contains normally conserved iron ligands (two His, one Asn, and one Ile residues, which is the protein’s C-terminal amino acid).

### 3.2 Gene expression of recombinant wheat LOX III and Mini-LOX III in yeast

**Fig. 2:**
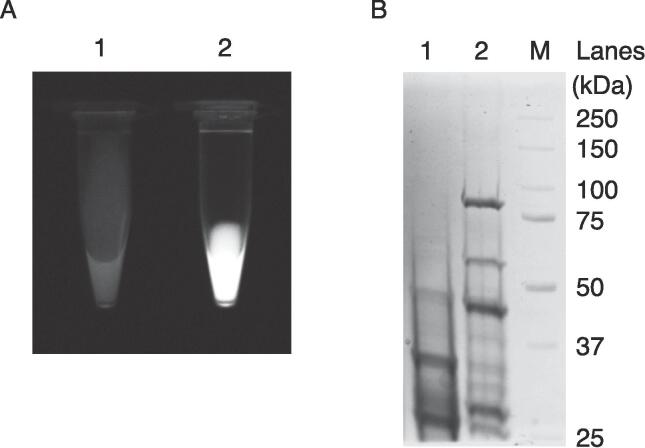
Expression of recombinant LOX III and mini-LOX III in yeast. (A) Fluorescence image of soluble extracts from yeast: soluble extract from recombinant LOX III-unexpressed yeast (Lane 1) and soluble extract from recombinant LOX III-2A-FP-expressed yeast (Lane 2). (B) SDS-PAGE analysis of the soluble extract from yeast: protein marker (Lane M), wild-type yeast (lane 1), and recombinant LOX III-expressing yeast (lane 2).

To assess the expression of recombinant wheat LOX III in yeast, we generated a reporter construct by linking the 2A peptide sequence derived from the equine rhinitis B virus between the upstream LOX III gene and the downstream fluorescent reporter mClover3 (Fig. S1A) (Souza-Moreira et al., 2018). LOX III and reporter genes were co-expressed in yeast via a bicistronic expression system. Recombinant LOX III and mClover3 were extracted from yeast cells by bead crushing. The presence of the reporter mClover3 in the soluble extract was visualized by fluorescence imaging, providing strong evidence for the expression of recombinant LOX III in the extract (Fig. 2A). The extracted proteins were subjected to 10 % SDS-PAGE and stained with CBB R-250. The gel indicated that the products of recombinant LOX III were approximately 88, 59, 41, and 27 kDa. Wheat LOX III is known to be cleaved by a reducing agent, such as 2-mercaptoethanol, to an N-terminus of approximately 40 kDa and a C-terminus of approximately 59 kDa (Andreou and Feussner, 2009, Feng et al., 2012). The products for recombinant LOX III were thus approximately 88 kDa for the polypeptide containing C-terminal LOX III, 2A peptide, and mClover3, 59 kDa for the polypeptide of C-terminal LOX III, 41 kDa for the polypeptide of N-terminal LOX III, and 27 kDa for the polypeptide of mClover3. The observed molecular weights aligned with the values predicted from the amino acid sequences (Fig. 2B). Based on the finding that recombinant LOX III can be expressed in yeast, a DNA construct containing the Mini-LOX III gene was generated (Fig. S1B), and recombinant Mini-LOX III was expressed in yeast, as described above (data not shown).

### 3.3 Activities of recombinant LOX III and Mini-LOX III on a linoleic acid substrate

**Fig. 3:**
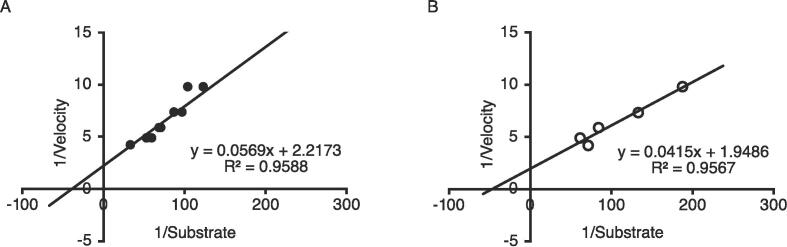
Activities of recombinant LOX III and Mini-LOX III on linoleic acid substrate. Lineweaver–Burk plots of the lipoxygenase activity of recombinant LOX III (A) and recombinant Mini-LOX III (B).

The Lineweaver–Burk method was used to determine the effect of substrate concentration on the initial velocities of reactions catalyzed by recombinant LOX III and Mini-LOX III extracted from yeast. Fig. 3A and B show the double-reciprocal plots of the initial velocities versus various linoleic acid concentrations. The Michaelis constant (Km) and the maximum velocity (Vmax) values for recombinant LOX III were determined to be 25.7 µM and 0.45 U/µL, respectively. For recombinant Mini-LOX III, the Km and Vmax values were determined to be 21.3 µM and 0.51 U/µL, respectively. These results showed that the C-terminal domain of wheat LOX III possesses catalytic activity.

### 3.4 Effect of recombinant LOX III isozyme on glutenin protein

**Fig. 4:**
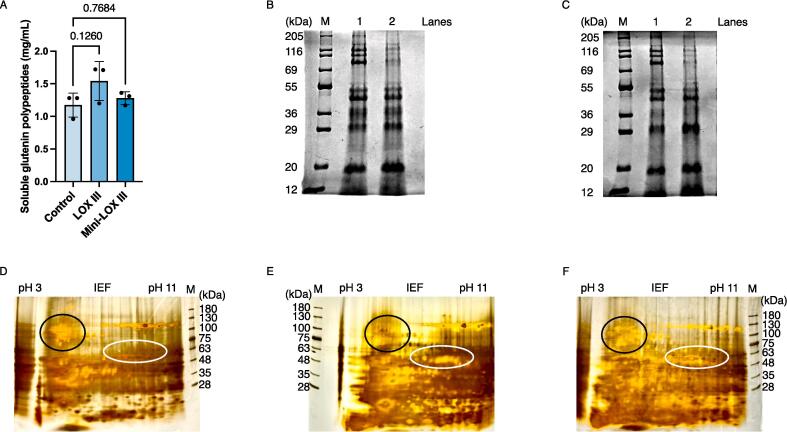
Effects of recombinant LOX III and Mini-LOX III on glutenin protein. (A) Amounts of soluble glutenin with and without recombinant LOX III or Mini-LOX III. The data represent the average of three independent experiments. Error bars represent the standard deviation (n = 3). One-way analysis of variance (one-way ANOVA) followed by Tukey’s multiple comparison test was performed using Prism 10 software (GraphPad Software, San Diego, California, USA) to produce p-values. (B) SDS-PAGE analysis of soluble glutenin components as follows: protein marker (Lane M), wild-type yeast (lane 1), and recombinant LOX III-expressing yeast (lane 2). (C) SDS-PAGE analysis of soluble glutenin components as follows: protein marker (Lane M), wild-type yeast (lane 1) and recombinant Mini-LOX III-expressing yeast (lane 2). (D) 2D-PAGE analysis of soluble glutenin components in wild-type yeast. (E) 2D-PAGE analysis of soluble glutenin components in recombinant LOX-III-expressing yeast cells. (F) 2D-PAGE analysis of soluble glutenin components in recombinant Mini-LOX III-expressing yeast cells. The major regions of different polypeptides with molecular weights of approximately 70–130 kDa and 55 kDa are black-circled and white-circled, respectively.

It has been suggested that natural LOX III purified from wheat germ might induce changes in the composition of glutenin subunits because of the reduced surface hydrophobicities of aggregative and low-molecular-weight glutenin subunits (Shiiba et al., 1991). Therefore, we evaluated the effect of adding recombinant wheat LOX III or Mini-LOX III extracted from yeast on glutenin subunit composition in wheat flour. The amounts of soluble glutenin in the dough with and without wheat recombinant LOX III or Mini-LOX III were 1.17 ± 0.18 g in control (without recombinant LOX III or Mini-LOX III), 1.54 ± 0.30 g with recombinant LOX III, and 1.28 ± 0.10 g with recombinant Mini-LOX III (Fig. 4A). These results indicated a slight increase in soluble glutenin.

To assess the polypeptide composition of soluble glutenin protein, sodium lauryl sulfate–polyacrylamide gel electrophoresis (SDS-PAGE) analysis was performed on solubilized glutenin proteins with and without recombinant LOX III or Mini-LOX III (Fig. 4B and C). Compared to soluble glutenin in dough without wheat recombinant LOX III or Mini-LOX III, soluble glutenin in dough exhibited a significant decrease in the amounts of polypeptides in the high-molecular-weight region above 70 kDa. On the other hand, polypeptide levels in the low-molecular-weight regions below approximately 40 kDa were significantly increased in the recombinant LOX III- or Mini-LOX III-treated soluble glutenin than in the untreated sample. Additionally, two-dimensional polyacrylamide gel electrophoresis (2D-PAGE) was performed on solubilized glutenin proteins with and without recombinant LOX III or Mini-LOX III (Fig. 4D-E). When recombinant LOX III and Mini-LOX III were treated with soluble glutenin, the amounts of polypeptide spots in the 70–130 kDa high-molecular-weight region with an isoelectric point at acidic pH decreased, whereas the amounts of polypeptide spots in the approximately 55 kDa mid-molecular-weight region with an isoelectric point at alkaline pH increased. These results indicate that the catalytic activities of recombinant LOX III and Mini-LOX III have an important effect on the compositional changes of glutenin proteins.

## 4 Discussion

LOX isozymes are produced in wheat germ, albeit in limited quantities. It is difficult to obtain large amounts of LOX from wheat germ, and the effect of LOX isozymes on wheat proteins is still largely unknown. In this study, we focused on wheat LOX III, which has a stronger effect on bread-making quality than LOX I or LOX II. We expressed recombinant LOX III in the heterologous host Baker’s yeast (*S. cerevisiae*) using a protein expression system. Recombinant LOX III expressed in yeast was extracted by bead crushing and used as a crude extract in this study. The utilization of yeast, an indispensable component of dough fermentation, ensures that crude extracts containing recombinant LOX III do not have deleterious effects on the secondary processing of flour. Recombinant LOX III activity was evaluated using the LOX enzymatic assay (Fig. 3A), and the Km value for recombinant LOX III was established to be 25.7 µM, with a Vmax of 0.45 U/µL. The Km values of LOX enzymes from various species obtained for linoleic acid were as follows: 0.24 mM for purified LOX from lupin seeds (Olias & Valle, 1988), 0.06 mM for purified LOX I and 0.18 mM for purified LOX II from barley (Hugues et al., 1994), 0.44 mM for purified LOX from pea seeds (Szymanowska et al., 2009), 2.33 mM for crude extracted LOX from fresh green pea (Gökmen et al., 2002), and 11.2 µM for purified LOX I from soybean (Maccarrone et al., 2001). It is difficult to compare the Km value of crude LOX extract with that of purified LOX. However, the present results suggest that the affinity of wheat recombinant LOX III for linoleic acid was higher than that of most other species of LOX. Compared to soybean LOX, which allows increased dough mixing tolerance (Hoseney et al., 1980), it is known that wheat LOX isozymes have differential physical properties in the dough (Shiiba et al., 1991). The differences in rheological effects between adding soybean LOX and wheat LOX III seem to be caused by their different substrate specificities. Soybean LOX attacks unsaturated triglycerides, free fatty acids, and monoglycerides, whereas wheat LOX isozymes mainly react with free fatty acids and monoglycerides (Veldink et al., 1977). This may explain why flour with wheat LOX III exhibited different rheological properties from flour with soybean LOX. These findings suggest that yeast-expressed recombinant LOX III can serve as an evaluative system for the processing of cereal-based foods.

Soybean LOX-1 has been widely used as a model system for studying the structural and functional properties of lipoxygenases from various species (Brash, 1999). Soybean LOX-1 comprises two domains: an N-terminal domain of approximately 30 kDa and a C-terminal domain of approximately 60 kDa, the latter of which harbors the catalytically active iron and the substrate-binding pocket (Maccarrone et al., 2001, Di Venere et al., 2003). In contrast, mammalian lipoxygenases do not have the N-terminal domain present in soybean LOX-1, wheat LOX, and other related plant lipoxygenases, thus showing smaller molecular masses (75–80 kDa compared to 94–104 kDa in plants). It has been suggested that the N-terminal domain in plant LOX only loosely contacts the C-terminal domain (Boyington et al., 1993), and that this domain may also be dispensable for plant lipoxygenases (Minor et al., 1996) because all the amino acid side chains responsible for catalysis are in the C-terminal domain. Maccarrone et al. (2001) discovered that mini-LOX possesses greater catalytic activity than intact soybean LOX-1. This finding suggested that the N-terminal domain has an intrinsic inhibitory function. Additionally, mini-LOX displayed a higher binding affinity for artificial membranes than intact LOX I, thereby indicating that the removal of the 30-kDa N-terminal fragment resulted in greater surface hydrophobicity (Di Venere et al., 2003). Similar results were reported in another study on the effect of N-terminal domain removal on the activity and membrane binding ability of rabbit reticulocyte-type 15-lipoxygenase (Walther et al., 2002). In the present study, recombinant Mini-LOX III activity was evaluated using a LOX enzymatic assay (Fig. 3B), and the Km value for recombinant Mini-LOX III was established to be 21.3 µM, with a Vmax of 0.51 U/µL. We determined that the activity of recombinant Mini-LOX III, which is an N-terminally truncated LOX III, was almost the same as that of intact recombinant LOX III. This finding suggests that the C-terminus of wheat LOX III contains a catalytically active domain and that recombinant Mini-LOX III has a strong effect on wheat proteins as intact recombinant LOX III.

To understand the role of wheat LOX III in the secondary processing of flour, we evaluated the effects of recombinant wheat LOX III and mini-LOX III on the polypeptide composition of soluble glutenin protein. The amount of soluble glutenin in dough with recombinant wheat LOX III or Mini-LOX III was slightly higher than in dough without them (Fig. 4A). The polypeptide composition of soluble glutenin in dough with recombinant wheat LOX III or Mini-LOX III resulted in significant changes compared to dough without recombinant wheat LOX III or Mini-LOX III (Fig. 4B and C). This result suggests that the catalytic activities of recombinant LOX III and Mini-LOX III, which oxidize linoleic acid, play important roles in changes in glutenin composition. In particular, the amounts of polypeptides decreased significantly in the high-molecular-weight region above 70 kDa, suggesting that soluble glutenin may shift insoluble. On the other hand, the amounts of polypeptides increased significantly in the low-molecular-weight range below approximately 40 kDa, suggesting that insoluble glutenin may be soluble. In the process of glutenin shifting from insolubility to solubilization, LOX III reacts with lipids (such as linoleic acid) that hydrophobically bind to glutenin. This reaction prompts lipids to form fatty acid hydroperoxides, which reduce the hydrophobicity of the glutenin protein surface. This phenomenon could explain the shift in glutenin composition toward increased solubility. This effect was probably more pronounced in the low-molecular-weight region of glutenin, where there are fewer glutenin molecules bound to lipids. However, oxidation by lipid peroxyl radicals generated in this reaction strongly induces disulfide bond formation in glutenin proteins (Zhang et al., 2012). This effect may occur in glutenin proteins in the high-molecular-weight region, where more glutenin molecules are bound to lipids. These conformational changes in glutenin proteins in the high-molecular-weight region probably induced a shift from soluble to insoluble. However, further studies are essential to elucidate the effects of LOX III and Mini-LOX III on the polypeptide composition of glutenin proteins. In detailed 2D-PAGE analysis (Fig. 4D-E), treating soluble glutenin with recombinant LOX III and Mini-LOX III, the amounts of polypeptide spots decreased in the high-molecular-weight region of 70 to 130 kDa, which has an isoelectric point at acidic pH, but increased in the medium-molecular-weight region, approximately 55 kDa, which has an isoelectric point at alkaline pH. Although new insights into the shift between soluble and insoluble in specific polypeptide spots were demonstrated in this study, the glutenin polypeptides that are sensitive to wheat LOX III remain unclear. This highlights a significant challenge for future research to precisely identify and characterize glutenin polypeptides that respond to wheat LOX III activity. Further studies are required to elucidate the specific interactions and mechanisms by which LOX III influences the solubility and functionality of glutenin polypeptides in wheat. Addressing these challenges will not only deepen our understanding of wheat biochemistry, but also potentially reveal new avenues for improving wheat quality and processing characteristics.

## 5 Conclusion

Our study presents significant advancements in the understanding of the role of wheat LOX III in the secondary processing of flours. The successful expression and characterization of recombinant wheat LOX III and Mini-LOX III in yeast is a crucial step in exploring their potential industrial applications. These recombinant enzymes exhibited distinct kinetic properties and demonstrated catalytic activity toward linoleic acid substrates, indicating their functional efficacy. Additionally, the application of these recombinant enzymes to wheat flour revealed the effects of glutenin protein composition. In particular, the addition of recombinant LOX III and Mini-LOX III resulted in an increase in the amount of soluble glutenin and a shift in polypeptide composition, with a significant decrease in the high-molecular-weight region and a significant increase in the low-molecular-weight region. These results suggest that the catalytic activities of recombinant LOX III and Mini-LOX III, which oxidize linoleic acid, play important roles in changes in glutenin composition. These conformational changes in glutenin proteins following treatment with recombinant LOX III and Mini-LOX III potentially affected dough properties.

Our findings suggest that yeast-expressed recombinant LOX III and Mini-LOX III can serve as effective tools for altering the polypeptide composition of glutenin in dough, which holds promise for improving the quality of cereal-based foods. These insights contribute to a deeper understanding of the enzymatic processes involved in cereal food production, and offer new avenues for enhancing the textural and quality attributes of flour-based products. In conclusion, this study highlights the potential use of recombinant wheat LOX isozymes in the food industry, particularly in the processing of cereal foods. The ability to manipulate glutenin composition through enzymatic intervention opens up possibilities for tailored food-processing techniques aimed at achieving the desired dough characteristics and product quality.

## Funding and additional information

This research did not receive any specific grants from funding agencies in the public, commercial, or not-for-profit sectors.

## CRediT authorship contribution statement

**Shunsuke Takahashi:** Conceptualization, Investigation, Methodology, Visualization, Writing – original draft, Writing – review & editing. **Gao Yue:** Investigation, Methodology, Visualization. **Reina Miyagi:** Methodology, Visualization. **Shiiba Kiwamu:** Supervision, Writing – review & editing.

## Declaration of competing interest

The authors declare the following financial interests/personal relationships which may be considered as potential competing interests: Shunsuke Takahashi and Kiwamu Shiiba has patent pending to Tokyo Denki University.

## Data Availability

The authors do not have permission to share data.
