# Supplementary material for: Production of recombinant intact and N-terminal truncated lipoxygenase isozyme III expressed in Saccharomyces cerevisiae and its influence on glutenin polypeptides
Source: Food Chem (Oxf). 2024 Jan 30;8:100195. doi: 10.1016/j.fochms.2024.100195 (PMC10847848; doi:10.1016/j.fochms.2024.100195)
Supplement: Supplementary data 1 [file mmc1.pdf]

# Production of Recombinant Intact and N-Terminal Truncated Lipoxygenase Isozyme III Expressed in *Saccharomyces cerevisiae* and Its Influence on Glutenin Polypeptides.

## *-Supplementary Information-*

Shunsuke Takahashi*, Gao Yue, Reina Miyagi and Shiiba Kiwamu*

Division of Life Science and Engineering, School of Science and Engineering, Tokyo Denki University, Ishizaka, Hatoyama-cho, Hiki-gun, Saitama, 350-0394, Japan

*Corresponding author. Email: stakahashi@mail.dendai.ac.jp, shiibak@mail.dendai.ac.jp

| | |
|---|---|
| **Supplementary Figure 1** | ***Page 2*** |
| **Supplementary Table 1** | ***Page 3*** |
| **Supplementary Table 2** | ***Page 4*** |
| **Supplementary Table 3** | ***Page 5-6*** |

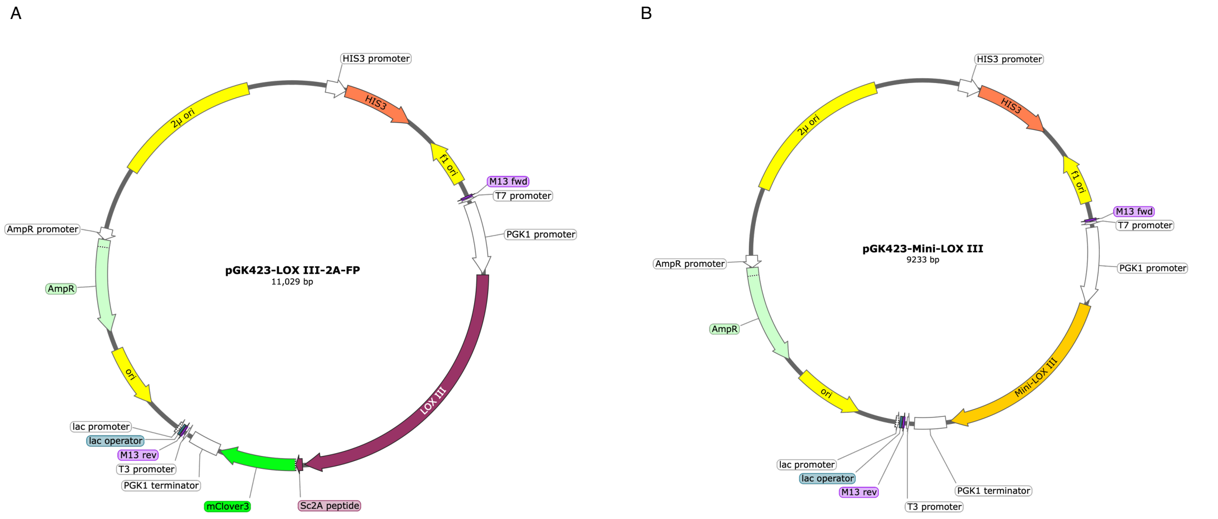


**Supplementary Figure 1 | Illustration of a series of LOX III expression vectors.** Map of (A) pGK423-LOX III-2A-FP and (B) pGK423-Mini-LOX III vectors.

**Supplementary Table 1 | List of plasmids used in this study.**

| Plasmids | Description | Reference |
|---|---|---|
| pGK423 | HIS3 marker, AmpR, PGK1 promoter system, 2μ origin, pBR322 origin (7.6 kb) | Ishii et al., 2009 |
| pGK423-2A-FP | pGK423 derivative, containing 2A self-cleaving peptides and mClover3 under PGK1 promoter | This study |
| pGK423-LOX III-2A-FP | pGK423 derivative, containing LOX III, 2A self-cleaving peptides and mClover3 under PGK1 promoter | This study |
| pGK423-Mini-LOX III | pGK423 derivative, containing Mini-LOX III under PGK1 promoter | This study |

**Supplementary Table 2 | List of primers used for molecular cloning.**

| Name | Nucleotide sequence (5’-3’) |
|---|---|
| LOX III SalI Fw | ATGATGGTCGACATGTTATTACATGGTTTGGTTGATAGGTTG |
| LOX III BamHI Rv | CATCATGGATCCATGATACTGACTCGA |
| PGK423 Fw | TAGTTTTTCAAGTTCTTAGA |
| PGK423 Rv | CTATTATTTTAGCGTAAAGG |
| LOX III SEQ001 | AAGAGGTGATGGTACCACCGG |
| LOX III SEQ002 | CCGTGTACGGTGATCAATCCTC |
| LOX III SEQ003 | CAGTACCAGATTCCTCCAGTCC |
| LOX III SEQ004 | AGGACCCTGCATTGAAGAACAGG |
| Mini-LOX III SalI Fw | ATGATGATGGTCGACATGGTTGCCGGGGACGACTTG |
| Mini-LOX III BamHI Rv | CATCATCATGGATCCATTCAGATACTGACTCGATTTGGAATTCCCATAGC |
| PGK423 2A-FP Gibson Fw | AACGCTAGCGTCGACACTAGTGGATCCCCCGGGTCTAGAGGGTGCTACTAATTTTTCTTTGTTGAAATTGGC |
| PGK423 2A-FP Gibson Rv | ATCTATCGATTTCAATTCAATTCAATTTATTTCAGATCTGCTTGTACAGCTCGTCCATGCC |

**Supplementary Table 3 | Codon-optimized gene sequences.**

| Optimized sequence of LOX III |
|---|
| ATGTTATTACATGGTTTGGTTGATAGGTTGACTGGTAAAAATAAACAAGCTTGGAAAGAA<br>GGTAAAATCCGTGGTACCGCTGTTTTAGTTAAAAGTGATGTCTTAAACCTGGGTGATTTTC<br>ATGCGTCATTGCTTGATGGTGTGCATGATATTCTAGGAAAAGATGATGGGGCCATTTTCCA<br>TTTGGTGTCCGCGACCGCACCTGACCCACAAAATCCAAGAAGAGGGAAAGTTGGTAAGCC<br>AGCACACTTGGAGGAGATGGTTGTTACAATGAAAAGTAAGGCCGCAGGCGAATCAGTATT<br>TAAAGTTACCTTCGAATGGGATGACTCACAAGGAATACCAGGTGCTGTTGTAGTAAGAAA<br>TACATACCGCAGTGAATATCTATTGAAAACACTTACCTTACATGGTGTCCCTGGTAAAGGG<br>ACAGTAGTCTTCGTTGCCAATTCATGGATTTATCCAAACGTCGATAGAGTTTTTTTGCCA<br>ATGATACGTATTTGCCGTCGAAAATGCCAGCATTACTGGTCCAATACAGACAGGACGAAT<br>TGAACAATTTAAGAGGTGATGGTACCACCGGAAAATATGAAGAATGGGATAGAGTTTATA<br>GGTACGACTATTATAACGATTTGGGCGAACCTGATAAGGGTCATCCTAGGCCAGTACTCG<br>GTAGCACCCAAGAACTACCATATCCAAGGAGGTGTAGAACAGGTAGACCACCAACAAAG<br>ACCGACCCAAGGTCAGAATCCAGAATCCCACAGTATAAAATCCAAGAAGCTTTGAACATT<br>TATGTGCCACGTGATGAACGTTTTGGGCACTTGAAACTGAGCGATTTCCTGGGATATTCAC<br>TTAAAGCAATCACCGAAGCAATCCTGCCAGTTATTCGTACTTACGTCGATACGACGCCAA<br>AGGAATTCGATTCGTTCCAGGATATTTATGACTTGTACGACGGGCTCTTGAAAGTGCCTGA<br>CAATCAGCACTTGAAAGAACTAAAGAAGAAAATACCTTTGCAATTCATAAAATCGTTACT<br>ACCGGTTGCCGGGGACGACTTGTTGAACTTACCTCTACCACATGTAATCAGGTCAAACGA<br>CTATGCATGGAGGTCGGATGAGGAATTTGCAAGAGAAATGTTAGCAGGCGTTAATCCCGT<br>TTGTATCAAGCGCCTAACCGAATTCCCTGTCAAGTCAACTCTGGACCCATCCGTGTACGGT<br>GATCAATCCTCAACTATAACAGAAGACCAGATTCAGCAGAATCTGGAAGAAGGACTAACT<br>GTTAAGCAAGCAATGGAAAAAAATAGACTATTTATACTGGATCATCACGACAATTTTATG<br>CCATTTCTTGACCGTATAAATAAGTTGGAGGGTAATTATATCTATGCCTCAAGGACCCTAC<br>TCTTTCTGAAGGCTGATGGTACCCTTAAGCCGTTAGCTATCGAATTGAGCTTGCCTCACCC<br>AGATGGTATCCAACACGGGGCTAAGTCAACGGTGTACCTCCCAGCCGACATTGACTCGGG<br>TGTTGATGGTCAAATTTGGCAGCTAGCCAAAGCTTACGCTTCAGTGGATGATTCCGCATGG<br>CATCAACTAATCAGTCATTGGCTGAATACTTTGGCTGTTATTGAGCCGTTCGTGATTGCAA<br>CTAACAGGCAACTTTCAGTGGTACACCCCGTTCACAAACTTTTGAGACCACATTATCGGGA<br>CACATTAAATATAAATGCTTTAGCTAGAACGACATTGATCAATGCGGGGGGAGTCTTTGA<br>AATGACAGTATTCCCTGAAAAGTATGCACTGGAAATGAGTAGTATCGTGTATAAAAACTG<br>GAAATTAACTGAACAAGGATTGCCAGATGATTTAGTGAAAAGAGGTATGGCAGTACCAGA<br>TTCCTCCAGTCCATAcGGAGTTAGATTACTTATAAAAGACTACCCATACGCAGTGGATGGT<br>CTGGTTATTTGGTGGGCTATTGAGAGATGGGTTAATGAATACCTGGCCATATACTACCCTA<br>ATGATGGTGTATTGCGTGCCGACAAGGAATTAGAAGAATGGTGGAAAGAAGTCAGGGAA<br>GTTGGGCATGGTGACTTAAAAGACGCAGACTGGTGGCCTAAGATGGTAACTGTACAAGAA<br>CTGGCCAAGACATGCACAACCATTATATGGGTGGCCAGTGCGTTGCACGCTGCTGTAAAT<br>TTTGGTCAGTACCCTTACGCTGGTTACTTGCCTAATCGGCCCACTGTTTCCAGACGAAAGA<br>TGCCAGAAGAAGGTGAAGAAGAGTATAAGCAATTACAGAAAGGCGGTAAAGAAGCCGAT<br>AAAGTTTTTATCCACACTATTACGTCTCAATTTCAAACCATCCTAGGTATTACCCTGATCG<br>AAATTCTAAGCAAACATTCATCTGACGAAGTCTATTTAGGTCAGCGTGATACACCAGAAT<br>GGACCTCTGATGCAAAAGCTCTTGAAGCTTTTAAGCGGTTCGGTACCAGGTTGATGGAAA<br>TTGAGAAGAGAATCTTGGACATGAATAAGGACCCTGCATTGAAGAACAGGAACGGTCCA<br>GTAAAGATGCCCTACATGTTACTGTATCCAAACACTTCTGATGCTGGCGGGGAAAAGGGT<br>TTAGGATTGACAGCTATGGGAATTCCAAATCGAGTCAGTATC |

| Optimized sequence of Mini-LOX III |
| --- |
| ATGGTTGCCGGGGACGACTTGTTGAACTTACCTCTACCACATGTAATCAGGTCAAACGACT<br>ATGCATGGAGGTCGGATGAGGAATTTGCAAGAGAAATGTTAGCAGGCGTTAATCCCGTTT<br>GTATCAAGCGCCTAACCGAATTCCCTGTCAAGTCAACTCTGGACCCATCCGTGTACGGTGA<br>TCAATCCTCAACTATAACAGAAGACCAGATTCAGCAGAATCTGGAAGAAGGACTAACTGT<br>TAAGCAAGCAATGGAAAAAAATAGACTATTTATACTGGATCATCACGACAATTTTATGCC<br>ATTTCTTGACCGTATAAATAAGTTGGAGGGTAATTATATCTATGCCTCAAGGACCCTACTC<br>TTTCTGAAGGCTGATGGTACCCTTAAGCCGTTAGCTATCGAATTGAGCTTGCCTCACCCAG<br>ATGGTATCCAACACGGGGCTAAGTCAACGGTGTACCTCCCAGCCGACATTGACTCGGGTG<br>TTGATGGTCAAATTTGGCAGCTAGCCAAAGCTTACGCTTCAGTGGATGATTCCGCATGGCA<br>TCAACTAATCAGTCATTGGCTGAATACTTTGGCTGTTATTGAGCCGTTCGTGATTGCAACT<br>AACAGGCAACTTTCAGTGGTACACCCCGTTCACAAACTTTTGAGACCACATTATCGGGAC<br>ACATTAAATATAAATGCTTTAGCTAGAACGACATTGATCAATGCGGGGGGAGTCTTTGAA<br>ATGACAGTATTCCCTGAAAAGTATGCACTGGAAATGAGTAGTATCGTGTATAAAAACTGG<br>AAATTAACTGAACAAGGATTGCCAGATGATTTAGTGAAAAGAGGTATGGCAGTACCAGAT<br>TCCTCCAGTCCATAcGGAGTTAGATTACTTATAAAAGACTACCCATACGCAGTGGATGGTC<br>TGGTTATTTGGTGGGCTATTGAGAGATGGGTTAATGAATACCTGGCCATATACTACCCTAA<br>TGATGGTGTATTGCGTGCCGACAAGGAATTAGAAGAATGGTGGAAAGAAGTCAGGGAAG<br>TTGGGCATGGTGACTTAAAAGACGCAGACTGGTGGCCTAAGATGGTAACTGTACAAGAAC<br>TGGCCAAGACATGCACAACCATTATATGGGTGGCCAGTGCGTTGCACGCTGCTGTAAATTT<br>TGGTCAGTACCCTTACGCTGGTTACTTGCCTAATCGGCCCACTGTTTCCAGACGAAAGATG<br>CCAGAAGAAGGTGAAGAAGAGTATAAGCAATTACAGAAAGGCGGTAAAGAAGCCGATAA<br>AGTTTTTATCCACACTATTACGTCTCAATTTCAAACCATCCTAGGTATTACCCTGATCGAA<br>ATTCTAAGCAAACATTCATCTGACGAAGTCTATTTAGGTCAGCGTGATACACCAGAATGG<br>ACCTCTGATGCAAAAGCTCTTGAAGCTTTTAAGCGGTTCGGTACCAGGTTGATGGAAATTG<br>AGAAGAGAATCTTGGACATGAATAAGGACCCTGCATTGAAGAACAGGAACGGTCCAGTA<br>AAGATGCCCTACATGTTACTGTATCCAAACACTTCTGATGCTGGCGGGGAAAAGGGTTTA<br>GGATTGACAGCTATGGGAATTCCAAATCGAGTCAGTATCTGA |

| Optimized sequence of mClover3 |
| --- |
| ATGGTGAGCAAGGGCGAGGAGCTGTTCACCGGGGTGGTGCCCATCCTGGTCGAGCTGGAC<br>GGCGACGTAAACGGCCACAAGTTCAGCGTCCGCGGCGAGGGCGAGGGCGATGCCACCAA<br>CGGCAAGCTGACCCTGAAGTTCATCTGCACCACCGGCAAGCTGCCCGTGCCCTGGCCCAC<br>CCTCGTGACCACCTTCGGCTACGGCGTGGCCTGCTTCAGCCGCTACCCCGACCACATGAAG<br>CAGCACGACTTCTTCAAGTCCGCCATGCCCGAAGGCTACGTCCAGGAGCGCACCATCTCTT<br>TCAAGGACGACGGTACCTACAAGACCCGCGCCGAGGTGAAGTTCGAGGGCGACACCCTG<br>GTGAACCGCATCGAGCTGAAGGGCATCGACTTCAAGGAGGACGGCAACATCCTGGGGCA<br>CAAGCTGGAGTACAACTTCAACAGCCACTACGTCTATATCACGGCCGACAAGCAGAAGAA<br>CTGCATCAAGGCTAACTTCAAGATCCGCCACAACGTTGAGGACGGCAGCGTGCAGCTCGC<br>CGACCACTACCAGCAGAACACCCCCATCGGCGACGGCCCCGTGCTGCTGCCCGACAACCA<br>CTACCTGAGCCATCAGTCCAAGCTGAGCAAAGACCCCAACGAGAAGCGCGATCACATGGT<br>CCTGCTGGAGTTCGTGACCGCCGCCGGGATTACACATGGCATGGACGAGCTGTACAAG |
